# Beyond printing: toward biologically informed biofabrication

**DOI:** 10.3389/fbioe.2026.1939524

**Published:** 2026-09-07

**Authors:** Antonella Motta

**Affiliations:** 1 Department of Industrial Engineering, University of Trento, Trento, Italy; 2 Department of Biochemistry and Microbiology, Faculty of Pharmaceutical Sciences, Chulalongkorn University, Bangkok, Thailand

**Keywords:** biofabrication, bioink, biological characterization, mechanobiology, regenerative medicine, regulatory science, traslational bioengineering

## Introduction

Over the last decade, biofabrication and bioprinting technologies have experienced remarkably rapid growth, emerging as some of the most dynamic and transformative areas in biomaterials and bioengineering research. Advances in additive manufacturing, biomaterial design, microfluidics, and cell-laden fabrication strategies have significantly expanded the ability to create increasingly complex and biologically relevant three-dimensional systems. As a result, biofabrication is now considered a key enabling technology for tissue engineering, regenerative medicine, advanced *in vitro* models, drug screening platforms, and personalized therapeutic approaches.

Initially driven largely by proof-of-concept studies demonstrating the possibility of organizing living cells and biomaterials into predefined architectures, the field is now progressively transitioning toward translational and clinically oriented applications. Increasing attention is being directed toward the development of functional tissues, scalable manufacturing strategies, and clinically compliant fabrication processes capable of supporting real biomedical applications.

However, together with this rapid technological evolution, an important challenge is emerging. Current research understandably places strong emphasis on engineering parameters such as printing precision, structural fidelity, manufacturing efficiency, scalability, and process reproducibility, all of which are essential for the technological advancement and translational reliability of biofabrication approaches. Nevertheless, these engineering aspects must be more closely integrated with a deeper understanding of the biological complexity of living systems.

When living cells are integrated into fabrication processes, biofabrication can no longer be regarded merely as a manufacturing technology. Instead, it becomes a highly dynamic biological process in which fabrication parameters directly modulate cellular behaviour, phenotype, mechanobiological responses, cell-cell communication, immune interactions, tissue maturation, and ultimately the long-term functionality and clinical performance of engineered constructs (Daly et al., 2021). Consequently, technological optimisation alone is insufficient if not accompanied by careful evaluation of the biological consequences induced during and after fabrication (Campbell et al., 2020).

In this context, cells cannot be considered passive building blocks merely embedded within printed constructs. During biofabrication processes, particularly in cell-laden bioprinting approaches, cells are exposed to multiple physical, mechanical, and chemical stresses—including shear forces, pressure gradients, nozzle-induced deformation, ultraviolet exposure during photopolymerization, thermal fluctuations, osmotic changes, and crosslinking conditions—that may significantly affect viability, phenotype, differentiation potential, mechanobiological responses, and long-term tissue functionality. Importantly, many of these effects are subtle and not immediately detectable through conventional short-term viability assays, while still profoundly influencing tissue maturation and functional outcomes over time (Blaeser et al., 2016; Nair et al., 2009).

One of the major limitations still present in part of current biofabrication research is the excessive reliance on short-term viability assays as primary indicators of biological success. High post-printing viability does not necessarily correspond to preserved cellular functionality. Cells may survive the fabrication process while undergoing significant alterations in gene expression, metabolic activity, mechano-transduction pathways, or immunomodulatory behaviour. In some cases, fabrication-induced stresses may trigger adaptive or inflammatory responses that only emerge after prolonged culture periods. As a result, constructs that appear structurally successful may still be biologically compromised.

Importantly, significant efforts are already being made by several research groups to address these limitations through more advanced and biologically comprehensive validation strategies, including transcriptomic and proteomic analyses, histological characterization, long-term functional assessment, and multi-scale biological evaluation. These approaches are contributing substantially to a deeper understanding of cell-material and cell-processing interactions. Nevertheless, such methodologies are not yet consistently integrated across the field and would benefit from broader adoption and standardization, particularly in view of translational and regulatory applications (Perin et al., 2026).

For this reason, the future development of biofabrication requires a stronger integration of biology into process design and optimization. Understanding how fabrication parameters influence cell behaviour at molecular, cellular, and tissue levels should become a central objective of the field. This includes the adoption of advanced biological characterization strategies, such as transcriptomic and proteomic analyses, mechanobiological investigations, long-term functional assays, and evaluations of tissue maturation and stability. The challenge is therefore no longer simply to fabricate increasingly complex structures, but rather to preserve and guide biological functionality throughout and after the manufacturing process (Formica et al., 2025; Zhang et al., 2026).

Addressing these issues inevitably requires a truly multidisciplinary approach. Biofabrication sits at the intersection of materials science, engineering, biology, physics, medicine, computational science, and regulatory disciplines. Yet these fields often operate with different priorities, methodologies, and languages. Engineers may focus on process precision and reproducibility, while biologists emphasize cellular responses and tissue functionality. Clinicians are primarily interested in safety, scalability, and translational feasibility, whereas regulatory experts require standardized validation and manufacturing consistency. Bridging these perspectives is essential if biofabrication technologies are to move beyond proof-of-concept demonstrations toward reliable clinical and industrial applications.

Within this framework, mechanobiology is likely to play an increasingly central role in next-generation biofabrication strategies (Zhang et al., 2025). Cells continuously sense and respond to mechanical cues from their environment, including stiffness, deformation, shear forces, and matrix architecture. The fabrication process itself therefore becomes part of the biological microenvironment. Designing biofabrication protocols that actively consider mechanobiological responses may significantly improve tissue maturation, functionality, and long-term stability. Similarly, the development of cell-instructive biomaterials capable of dynamically interacting with living systems represents an important step toward biologically informed biofabrication.

Another critical aspect concerns standardization, reproducibility, and scalability. Despite the rapid growth of the field, there is still a lack of harmonised methodologies for evaluating biofabricated constructs. Variability in cell sources, bioink formulations, printing parameters, and biological assessment protocols makes comparison between studies extremely difficult (Kesti et al., 2016). The establishment of standardized reporting criteria, biologically relevant validation frameworks, and manufacturing workflows compatible with industrial translation will therefore be essential for improving reproducibility and accelerating regulatory acceptance.

This becomes even more important considering the translational ambitions of the field. Clinical biofabrication applications will require not only technological sophistication, but also robust evidence regarding safety, functionality, manufacturing consistency, and long-term biological performance. Regulatory considerations should therefore not be viewed as external constraints introduced at late stages of development, but rather as integral components of the design process itself. At the same time, the emergence of increasingly complex biomaterials, hybrid living constructs, and advanced manufacturing technologies may require critical evaluation of whether current regulatory frameworks are fully adequate to address these innovations.

Looking forward, the future of biofabrication will likely depend on a conceptual transition from technology-driven fabrication toward biologically informed manufacturing. The central question should evolve from “Can we print complex living structures?” to “Can we preserve, control, and direct biological function during and after fabrication?”. Achieving this goal will require the integration of experimental biology, engineering, computational approaches, translational sciences, and regulatory awareness.

## Future directions

An additional challenge for the future of biofabrication concerns education and training. The increasing complexity of the field requires a new generation of scientists capable of operating across disciplinary boundaries, integrating expertise in biology, materials science, engineering, medicine, computational approaches, and regulatory frameworks. Traditional disciplinary training alone may no longer be sufficient to address the multifaceted challenges associated with the development and translation of biofabricated systems. Future educational strategies should therefore promote interdisciplinary environments, communication skills, translational awareness, and the ability to critically evaluate both technological and biological aspects of biofabrication processes. Training researchers able to bridge these different perspectives will likely become essential for the sustainable and clinically relevant evolution of the field.

In parallel, computational biology and artificial intelligence are expected to play an increasingly important role in accelerating process optimisation and reducing experimental burden REF. Predictive modelling, digital twins, machine learning-assisted biomaterial design, and computational simulations of cell-material interactions could significantly support the rational development of biofabrication protocols, enabling faster identification of optimal processing conditions while improving reproducibility and scalability. Such approaches may also contribute to reducing costs, minimizing biological variability, and facilitating translational validation.

Future strategies should also move toward a more inclusive, biologically representative, and societally relevant framework. Greater attention should be dedicated to variables such as age and sex, which remain significantly underrepresented in many experimental studies despite their well-recognised impact on cellular behaviour, tissue regeneration, immune response, and disease progression. The development of more sex- and age-aware biofabrication strategies, including female-oriented models and biomaterials, may substantially improve the physiological relevance and translational value of engineered systems.

Beyond regenerative medicine, biofabrication is also expected to play an increasingly important role in the development of advanced *in vitro* models for disease investigation, personalised medicine, and pharmaceutical screening. Patient-specific biofabricated systems may provide more physiologically relevant platforms for studying pathology progression, therapeutic responses, and inter-individual variability, potentially reducing the reliance on conventional animal models while improving the predictive value of preclinical testing. In this context, biofabrication technologies are progressively emerging as valuable tools for accelerating drug development and supporting precision medicine approaches.

At the same time, the impact of biofabrication is extending beyond biomedical applications. Emerging areas such as cellular agriculture and sustainable food production are beginning to exploit biofabrication strategies to engineer structured edible materials and cultured food products. These developments further highlight the multidisciplinary nature of the field and reinforce the need for scalable, reproducible, biologically informed, and sustainable manufacturing approaches capable of addressing both healthcare and broader societal challenges.

Finally, sustainability should become a central consideration in the evolution of biofabrication technologies. In alignment with emerging European strategies and regulatory frameworks focused on sustainable innovation and circular bioeconomy principles, the field should increasingly address the environmental impact of materials sourcing, manufacturing processes, energy consumption, waste generation, and supply-chain management. The development of scalable and sustainable biofabrication approaches, including renewable biomaterials, green processing technologies, and standardised manufacturing workflows, will likely become essential not only for industrial translation, but also for long-term regulatory and societal acceptance.

Biofabrication undoubtedly holds enormous potential for the future of regenerative medicine and advanced healthcare technologies. Nevertheless, the success of the field will ultimately depend not only on our ability to fabricate increasingly complex constructs, but also on our capacity to understand, preserve, and direct the biology embedded within them. Only through the integration of technological innovation with deep biological understanding, multidisciplinary collaboration, and translational awareness will biofabrication fully realize its clinical and societal promise.
